# Do social insects support Haig's kin theory for the evolution of genomic imprinting?

**DOI:** 10.1080/15592294.2017.1348445

**Published:** 2017-07-13

**Authors:** Mirko Pegoraro, Hollie Marshall, Zoë N. Lonsdale, Eamonn B. Mallon

**Affiliations:** Department of Genetics and Genome Biology, University of Leicester, UK

**Keywords:** Epigenetics, evolution, genomic imprinting, Haig's theory, social insects

## Abstract

Although numerous imprinted genes have been described in several lineages, the phenomenon of genomic imprinting presents a peculiar evolutionary problem. Several hypotheses have been proposed to explain gene imprinting, the most supported being Haig's kinship theory. This theory explains the observed pattern of imprinting and the resulting phenotypes as a competition for resources between related individuals, but despite its relevance it has not been independently tested. Haig's theory predicts that gene imprinting should be present in eusocial insects in many social scenarios. These lineages are therefore ideal for testing both the theory's predictions and the mechanism of gene imprinting. Here we review the behavioral evidence of genomic imprinting in eusocial insects, the evidence of a mechanism for genomic imprinting and finally we evaluate recent results showing parent of origin allele specific expression in honeybees in the light of Haig's theory.

## Introduction

Genomic imprinting is the differential expression of alleles in diploid individuals, with expression being dependent upon the sex of the parent from which the allele was inherited.^1^ Genomic imprinting is an evolutionary paradox. Natural selection is expected to favor expression of both alleles to protect against recessive mutations that render a gene ineffective.^2^ What then is the benefit of silencing one copy of a gene, making the organism functionally haploid at that locus? Several explanations for the evolution of genomic imprinting have been proposed.^3^

Haig's kinship theory, is the subject of this review. It is the most developed and best supported theory^3^ and the most applicable to social insects.^4^ Outside the scope of this review are several non-conflict based theories to explain the evolution of genomic imprinting that have been recently reviewed by Spencer and Clark.^5^

Haig's theory is based on the idea that maternally (matrigene) and paternally (patrigene) inherited genes in the same organism can have different selectional pressures (described in greater detail in the “Haig's kinship theory” section and Fig. 3). For example, in a species with multiple paternity, a patrigene has a lower probability of being present in siblings that are progeny of the same mother than does a matrigene. As a result, a patrigene will be selected to value the survival of the individual it is in more highly, compared with the survival of siblings. This is not the case for a matrigene. In mammals and angiosperms, this conflict is played out in the provisioning of offspring with resources taken from the mother.^2^ A patrigene will benefit by causing more maternal resources to be allocated to the individual it is in, but a matrigene will benefit from sharing resources among all the siblings. A method to differentiate matrigenes and patrigenes of these genes (i.e., imprinting) would spread in the population.

Haig's kinship theory is central to our evolutionary understanding of imprinting effects in human health and plant breeding;^6,7^ yet, despite its importance, the theory still lacks an independent test. Social insects have been suggested as such an independent test of Haig's kinship theory for the evolution of genomic imprinting.^4^ Here we review the behavioral evidence of genomic imprinting in social insects, evaluating recent results showing parent of origin allele specific expression in honeybees asking whether Haig's kinship theory predictions are supported. We also review the evidence for mechanisms in social insects for genomic imprinting in light of what is known in mammals. We then explore advances in technologies (e.g., next-generation sequencing, chromatin immunoprecipitation, CRISPR) that can be used in dissecting this mechanism genetically and molecularly. Finally, we propose a direction for future research suggesting a merger of genetic and molecular approaches to resolve pressing evolutionary and mechanistic questions on the nature of genomic imprinting.

### Genomic imprinting

Genomic imprinting is a phylogenetically widespread phenomenon, and genes have been shown to be imprinted in mammals,^8^ plants, and fungi,^9,10^ as well as insects,^11^ although see Coolon et al.^12^ In mammals, genomic imprinting is associated with both DNA and histone methylation and these epigenetic markers are established in the germline of parents and are preserved during development (see Box 1 Genomic Imprinting: the mammalian way).^13^

### Haig's kinship theory

Haig's kinship theory is an extension of the theory of inclusive fitness that was developed by W. D. Hamilton in 1964.^14^ The inclusive fitness theory postulates that the fitness of an individual is not solely dependent upon their own survival and reproduction, but it is also reliant on the survival and reproduction of those that share the same genes. Thus, an individual will favor actions that appear to be altruistic toward related individuals that will ensure shared genes are passed on to future generations.

Haig's kinship theory is currently the most widely accepted theory for the evolution of genomic imprinting.^5^ Developed in the late 1990s,^15^ Haig assumes that in a polyandrous mating system the genetic relatedness of the offspring is higher for maternally inherited alleles (matrigenes) than it is for paternally inherited alleles (patrigenes). Therefore, selection is expected to act on patrigenes to increase individual resource allocation from the mother, thus increasing paternal inclusive fitness. Whereas matrigenes should be selected to favor equal resource distribution among offspring to maximise maternal inclusive fitness.^16^ In this context the same genes may experience different evolutionary pressures depending upon whether they are in the parents or in their progeny (F1). Genes in the mother (or father) are referred to as **maternal** (or **paternal**) genes. Genes from the mother (or the father) in the progeny are called **matrigenic** (or **patrigenic**) and a **matrigene** (or a **patrigene**) refers to an allele in the progeny derived from the mother (or the father).

This theory explains for example how the cost-benefit ratio changes during sibling competition for parental resources (i.e., time, food).^4^ If there is no genomic imprinting and no difference in the coefficient of relatedness between patrigenes and matrigenes of offspring, an individual should weigh its own benefit (relatedness coefficient, r:1) against the cost for the sibling affected (full sibling, r:1/2; half sibling, r:1/4) and selfish or selfless behavior should not be particularly associated to patrigenes or matrigenes (Fig. 3). In a different situation where there is imprinting and the cost falls on siblings with a different father, the coefficient of relatedness is different between matrigenes (r:1/2) and patrigenes (r:0) (Fig. 3). In this situation patrigenes may be selected to be always selfish, while for matrigenes selfishness is advantageous only when the benefit is greater than 1/2 the cost to a sibling of a different father.

This theory has been used to explain why imprinting is prominent in angiosperms and mammals with extended parental care.^1,2^ The theory also provides an explanation to why many genes expressed in embryos, placenta, and sperm are imprinted, as well as why paternally and maternally imprinted genes have opposite effects on offspring size.^1,4^ Paternal genes are expected to favor larger offspring while maternal genes should favor smaller offspring in species with a polyandrous mating system that also provide maternal care.^1^ Larger offspring are able to elicit more resources from the mother, which is beneficial for the individual but detrimental for the siblings. This would be advantageous to patrigenes, that may not be present in all siblings, but disadvantageous to a matrigene that has a 50% chance of being present in the siblings. For example, inactivation of the paternal allele of the *Mest* gene in mice (maternally imprinted) produced smaller, lighter pups,^17^ giving weight to this theory. The function of most identified imprinted genes in mammals support this theory.^18,19^

The theory also extends beyond systems with known imprinting; Queller^4^ expands on Haig's original suggestions and applies the kinship theory to social insect colonies. The haplodiploid sex determination of eusocial insects creates a potential conflict of interest between patrigenes and matrigenes in a given offspring due to different degrees of relatedness with brothers and sisters (Fig. 3).^1,4^ Queller^4^ is able to make predictions for genomic imprinting based on various social contexts, for example queen competition and insertion of new queens, male killing, and worker reproduction. These predictions provide a solid ground with which to test the validity of the kinship theory and provide evidence for conflict within an individual genome.

### Do social insects possess the machinery for genomic imprinting?

Haig's kinship theory predicts imprinting for some genes in certain conditions, but this implies that the parents are able to differentially label their genes. How do they do that? Imprints are established in gametogenesis and embryogenesis, and are present in different tissues predominantly involved in the provision of nutrients to offspring. In mammals for example, most of the identified imprinted genes are expressed in the placenta.^20^ Moreover, the majority of imprinted genes in the angiosperm lineage are expressed in the endosperm which surrounds the plant embryo and is primarily a source of starch, but also oils and proteins.^21^ Genes have also been reported to be imprinted in the mammalian brain,^22,23^ and potentially in the angiosperm embryo too [see;^24^ however also see^25,26^].

Although much is still not understood, many molecular components of the mechanism of genomic imprinting (e.g., DNA methylation, histone modifications) are well studies in mammals and in plants (for recent reviews of gene imprinting mechanism in plants see^27,28^). Below we review the evidence for the presence of these imprinting mechanism in eusocial insects [see^29^].

### DNA methylation

**Figure 1.**
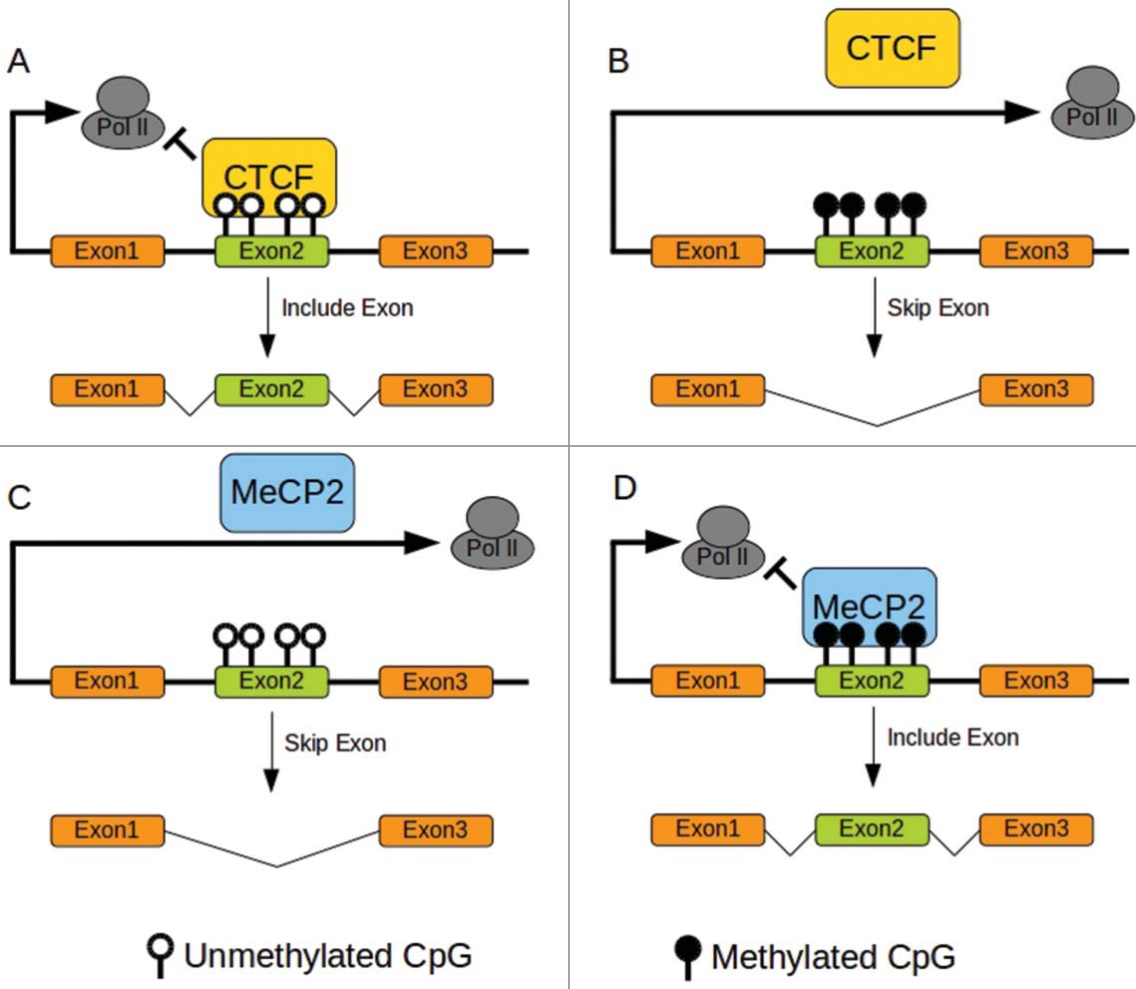
DNA methylation and splicing in mammals. (A)CTCF Binding to unmethylated CTCF binding sites pause Pol II elongation allowing retention of exon2. (B)CTCF cannot bind to methylated sites resulting in skipping exon2. (C)MeCP2 does not bind to unmethylated sites allowing rapid progression of Pol II resulting in skipping of exon2. (D)MeCP2 binding to methylated site pause elongation of Pol II permitting retention of exon2. Redrawn from Yan et al. 2015.^30^ CTCF binding site with methylation sensitive CpG in bold: ATGCAGCTAGATGG**CG**CTC.^74^

Among the epigenetic markers, the methylation of the fifth carbon of cytosine (5mC) is found to be associated with control of transcription and regulation of splicing^30^ (see Box 1, Genomic Imprinting: the mammalian way and Fig. 1).

DNA encoding for the DNA methyltransferases (DNMTs) and 5mC have been found in many hymenopteran species.^31^ In mammals, distinct classes of DNMTs are responsible for maintaining methylation through cell division (DNMT1) and de novo methylation (DNMT3s),^32^ but the insect catalytic activities of these enzymes have not been formally characterized. Some species, like the silkworm *B. mori*, have only a single DNMT with dual DNMT1/3 functions.^33^ The fruit fly, *D. melanogaster*, has lost both DNMT 1 and 3 and encodes for a DNMT2 enzyme that is involved in RNA methylation.^33^ Hymenoptera maintain all 3 classes of enzymes (DNMT1, 2, and 3) in single or multiple copies.^31,33-36^ The hymenoptera methylation toolkit also includes 10–11 translocation enzymes (TET) that catalyze the oxidation of 5mC to 5-hydroxymethyl cytosine (5hmC).^37^ This enzyme and 5hmC are thought to be part of the process of demethylation in mammals^38^ and may also have similar roles in insects.

The distribution of DNA methylation in hymenoptera shows similarities as well as differences with that of mammals. In mammals more than 70% of CpGs are methylated and methylation can be found in promoters as well as gene bodies,^39^ while in hymenoptera less than 2% of CpGs are methylated and are primarily localized in gene bodies.^31,35,40^

In mammals, whole genome assays uncovered a substantial difference between isolated CpG (usually heavily methylated) and hypomethylated grouped CpGs (CpG islands or CGIs: region of 500–2000 bp).^41^ A long held view associates DNA methylation with transcriptional repression, but deep analysis of CGIs and intragenic methylation began to erode this classic view. CGIs tend to correlate with promoter regions and can be classified as High, Intermediate or Low CpG density promoters (HCPs, ICPs, and LCPs respectively). HCPs are linked to transcriptionally active genes and increases in methylation indeed result in gene silencing. ICPs are also inactive when methylated, although the level of methylation changes significantly during differentiation.^42,43^ In contrast, LCPs are usually hypermethylated and transcriptionally active.^42,44^ Moreover, active genes in the inactive imprinted X chromosome, show a high level of gene body methylation. This could simply be a consequence of the transcription availability since in this case the genes would also be accessible to the DNA methylases,^45^ or the higher level of methylation may be related to RNA transcription and/or splicing given that the pattern of methylation marks intron-exon boundaries.^46^

In eusocial insects, the caste system imposes another layer of organization to the reprogramming and cell lineage specification that may be controlled by epigenetic mechanisms. It is possible that like different cell lineages acquire a particular epigenome during development, also different castes are epigenetically defined during development.^47,48^ There may be programming and re-programming windows during development and later in life, as for example suggested by a study in honeybee (*A. mellifera*) where phenotypic plasticity between nurses and foragers correlated with variation in DNA methylation in the brain.^49^ However, re-programming outside these coding windows may be difficult and result in less than fully penetrating phenotypes.^50,51^

Larvae, during development, may be considered dual potent because they retain the ability to develop into different castes (e.g., queens vs. workers) and many epigenetic tools (DNA methylation, miRNA, piRNA, etc.) as well as alternative splicing were implicated in this plasticity in the honeybee *A. mellifera* and to a lesser extent in the ants *Camponotus floridanus* and *Harpegnathos saltator*.^33-35,52^ Interestingly, DNMT1a is maternally transmitted to the progeny in the wasp *N. vitripennis* and its downregulation results in a block of development 10–12 h after egg laying, while DNMT3 is not essential for early embryo development.^53^ Similarly in mice, deletion of DNMT1 is lethal at gastrulation resulting in a global loss of DNA methylation.^54^ Differences in behavior in adult castes may be also associated with a difference in epigenetic programming. The role of DNA methylation in this context was recently tested with contrasting results in the honeybee *A. mellifera*, the ants *C. floridanus, H. saltator, Solenopsis invicta*, and *Ooceraea biroi*.^31,35,49,55,56^ Moreover, the function of DNA methylation is reported to be of less importance in the simple eusocial societies of the wasp *Polistes canadensis* and the ant *Dinoponera quadriceps* where these species are more reliant on transcriptional network re-organization to determine phenotype differences, in comparison with highly eusocial insects.^57^

Libbrecht et al. 2016, questioned the results of previous studies reporting caste dependent differences in DNA methylation, suggesting that they used a false positive-prone method in their next-generation sequencing data analyses.^55^ However, using methylation-sensitive AFLP, Amarasinghe and colleagues^58^ also found differences in methylation in the heads of reproductive and non-reproductive workers in queenless *B. terrestris* colonies. Drug manipulation of the methylation level clearly correlated DNA methylation with changes in phenotype/behavior of the workers. Importantly, this data support Haig's kingship theory predictions that genomic imprinting should be important in worker reproductive behavior. The theory predicts in fact that there should be conflict between maternally and paternally derived alleles in loci involved with workers reproduction resulting in genomic imprinting^4^ (see section: Parent of origin allele specific expression).

Whether differences in DNA methylation are hallmarks of different caste specific behavior is still to be resolved. It is worth noting here that DNA methylation plays an important role in learning and memory formation in both mammals (rats) and honeybees and is dynamically regulated in mammalian adult brains.^59,60^

### Gene body methylation and splicing

CpG methylation in gene bodies seem to have a conserved function between mammals and hymenoptera. CpG methylation was found mostly in exons marking intron-exon boundaries and was associated with controlling splicing in the honeybee *A. mellifera*,^34,49,61,62^ in the wasp *N. vitripennis*^63^ (but see also^40^) and in both the ants *C. floridanus* and *H. saltator*.^35^ Highly methylated genes were found to be uniformly and transcriptionally active in different conditions and representing housekeeping genes expressed in most cell types.^34,35,40,64,65^ This also holds true in a social insect outside of hymenoptera, the termite *Zootermopsis nevadensis* (isoptera) shows the same intragenic methylation associated with highly expressed genes and alternative splicing.^66^ In contrast, sparsely methylated genes are associated with caste-specific gene expression in germ lines in honeybees.^67^ How can DNA methylation mediate splicing and even participate in the process of alternative splicing? Recent data from mammals may provide some clues.

DNA methylation can change the availability of a locus to a protein or a protein complex. This is the case of ZFP57, a protein important to maintaining genomic imprinting during development in mammals (see Box 1: Genomic Imprinting: the mammalian way). ZFP57 affinity for its binding site increases with methylation.^68^

Another example is the allele-specific expression of H19/IGF2 locus. The allele-specific level of methylation of the imprinting control region (ICR) in this locus mediates the binding of CCCTC-binding factor (CTCF) that in turn controls the expression of H19 (maternal allele) or IGF2 (paternal allele).^69^ The binding of CTCF is a pausing signal for RNA polymerase II (Pol II). DNA methylation inhibits CTCF binding, therefore enabling Pol II elongation resulting in skipping of an exon (Fig. 1).^70^ This mechanism could explain why hypomethylation was associated with alternative gene splicing in the gene *alk* in the honeybee *A. mellifera* and *lipoprotein receptor 2* in the ant *C. floridanus*.^71,72^

Conversely, the mammalian methyl-CpG-binding protein 2 (MeCP2) is involved in exon retention, but mirrors CTCF in its affinity to DNA methylation (Fig. 1).^73^ MeCP2 has greater affinity for its methylated binding site inducing a pause in Pol II elongation and retention of the target exon (Fig. 1). High levels of methylation were associated with retention of exons in the ants *C. floridanus* and *H. saltator*^35^ that could be achieved via a MeCP2 like mechanism. These 2 mechanisms (MeCP2 like, CTCF like) could be operating in concord, resulting in a complex relationship between DNA methylation and control of alternative splicing.

It is important here to note that MeCP2 was implicated in neuronal function that in mammals relies heavily on alternative splicing.^75^ Similarly in *A. mellifera* the transition from nursing to foraging associates with alternative splicing events.^49^ In the plant *Arabidopsis thaliana* and in the green spotted puffer-fish *Tetraodon nigroviridis* a particular histone variant (H2A.Z) is enriched in hypomethylated loci and could serve as a marker for alternative splicing^76,77^ while in mammals the methylation of H3K9 by the Histone H3K9 methyltransferase G9a recruits DNMTs to unmethylated loci and changes chromatin status.^78^ Although little evidence is at the moment available, it has been shown that histone deacetylases (HDACs) in the ant *C. floridanus* are involved in the transition to foraging/scouting suggesting a possible role for histone modifications in determining caste-specific patterns of behavior.^79^ It is probable that DNA methylation is therefore acting in concert with other epigenetic modifiers (e.g., ncRNAs, histone modifications) to regulate alternative splicing in hymenoptera^71,80^ in a cell specific manner.

Further investigation is needed to reveal the mechanistic role of DNA methylation and other epigenetic modifications in regulating alternative splicing in hymenoptera.

### Other epigenetic mechanisms

Regardless of the role of DNA methylation, other epigenetic markers could be important in defining a specific caste related behavior. Long non-coding RNA (lncRNA) and microRNA (miRNA) are potential additions to the epigenetic tool kit in defining behaviors. miRNA may have a role in determining caste-specific expression profiles in *A. mellifera*, targeting unmethylated genes during development.^81^

Changes in miRNA expression profiles are also associated with the nurse-forager transition.^82^ Another class of small RNA (piRNA, PIWI interacting RNA) are associated to genomic imprinting of the paternally methylated *Rasgrf1* in mammals.^83^ In addition, some lncRNA have being implicated in mediating aspects of brain development, functional diversification (*kakusei*,^84^), and the transition from nursing to foraging (Nb-1,^85^) in *A. mellifera*. In mammals, lncRNA are also related to genomic imprinting (for recent reviews see^86^ and ^87^). All clusters of imprinted genes in mammals include one or more lncRNA^88^ and the genes and the lncRNA in these clusters are often expressed from the opposite parental chromosomes.^89^ lncRNA use a variety of molecular strategies to epigenetically regulate transcription of imprinted clusters.^86,87^

**Figure 2.**
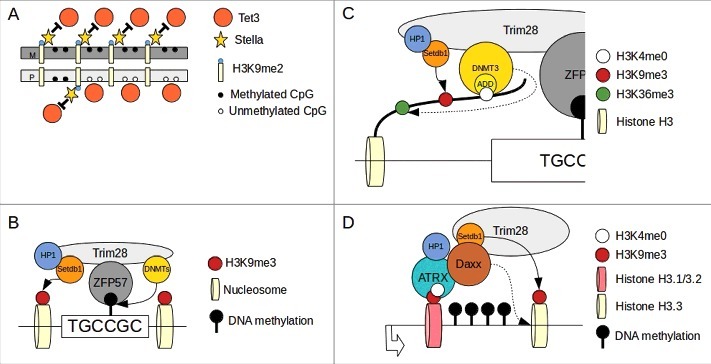
Mechanism of gene imprinting in mammals. (A)Stella binding to H3K9me2 prevent TET3 dependent de-methylation. Maternal (M) DNA is enriched of H3K9me2 compared with paternal (P) and therefore maternal imprinted regions are protected from active de-methylation. (B)After zygote formation and during the firsts cell divisions, level of methylation at imprinted loci is maintained by ZFP57/TRIM28 complex binding to methylated consensus and recruitment of DNMTs. (C)DNMT3a/b interact with H3 tail via ADD domain and their activity is permitted only when H3K4 is unmethylated (H3K4me0). This modification is enriched in the DNA methylated allele of imprinted loci (see text). DNMT3s interact also via a PWWP domain (dotted arrow) to H3K36me3 (enriched in gene bodies of active genes). H3K9me3 methylation is maitained by Setdb1 (continuous arrow). (D)At transcriptionally active imprinted loci the S-phase specific H3.1/H3.2 histones are exchange for the cell cycle independent H3.3 histone. The ATRX/Daxx complex is responsible for the exchange of H3.1/3.2 with H3.3 (dotted arrow). ATRX bind H3K4me0 via an ADD domain and interact also with to H3K9me3. Daxx recruit Setdb1 that maintain H3K9me3 methylation (continuous arrow). Re-draw from Messerschmidt et al. 2014 and Voon and Gibbons 2016^8,91^

More work is required to reveal how the different epigenetic markers are intertwined together to generate the different pattern of behavior and expression, genomic imprinting during gameto- and embryogenesis resulting in phenotypic plasticity in hymenoptera.

It seems that even the variant of histone allocated to imprinted loci contributes to maintaining imprinting. During development the H3.3 histone variant was found to be enriched in heterochromatic regions including the DNA methylated allele in imprinted loci.^125,126^ The chaperone responsible for the allocation of H3.3 in this region is the ATRX/Daxx complex that preferentially targets the methylated allele.^125,126^ ATRX has an ADD domain to interact with H3K4me0 and can also directly bind H3K9me3 and HP1.^127^ It has been shown in addition that Daxx interacts with TRIM28 and Setdb1.^128^ So the ATRX/Daxx complex could represent another way (alternatively or in combination with ZFP57) to recruit TRIM28/Setdb1 to imprinted regions and maintain DNA methylation during development (Fig. 2).

Whether such an exquisite epigenetic reprogramming with successive waves of de-methylation and re-methylation during gametogenesis and embryogenesis exists in hymenoptera, is not currently known. The role of histone modification is also unknown. In honeybee at the present time there is no evidence for a comprehensive de-methylation program during embryogenesis.^129^ Moreover, methylation levels are retained through early stages of development, as they also are in other animals (e.g., *Danio rerio, Caenorhabditis elegans*) although the specific mechanism is still to be determined.^130,131^

Box 1. **Genomic Imprinting: The mammalian way**

The expression of imprinted genes in mammals is coordinated by allele-specific (maternal or paternal) DNA methylation in imprinting control regions (ICRs).^90^ Allele-specific methylation or parent-of-origin-specific methylation is introduced during gametogenesis and it is preserved throughout the entire life. During gametogenesis in fact, the somatic epigenetic code is removed in the primordial germ cells (PGCs). A new sex specific and germ specific epigenetic code and transcription profile is established and finally a post-fertilization removal of this epigenetic code is activated to allow further embryonic development.^91^ The ICRs allele specific methylation therefore must be protected from the post-fertilization wave of de-methylation.

The first wave of de-methylation in the PGCs erases the somatic epigenetic code and establishes totipotency. The global level of DNA methylation at this stage is dramatically reduced and parental imprinting and X-chromosome silencing are removed in mouse embryos.^92^ This reprogramming is achieved trough a combination of passive and active de-methylation. During the PGCs proliferation the de-novo DNMT3s are repressed and Np95 (an essential cofactor for DNMT1) is either downregulated or excluded from the nucleus.^92,93^ This passive de-methylation affects the genome globally, but some regions (including imprinted genes, CGIs of inactive X-chromosomes) are hypomethylated only after an active de-methylation involving TET1/2 and 5hmC.^92,94^

Following this PGC re-programming, a new sex-specific epigenetic code and ICR methylation is established. The exact mechanism of this precise re-methylation is still to be uncovered, but it appears to be related more to the chromatin structure, histone modifications and transcription factors, as opposed to sequence specificity.^95,96^

The second wave of epigenetic reprogramming takes place post fertilization during early embryogenesis and shows important differences to the quasi total de-methylation occurring in PGCs. The kinetics of de-methylation of the zygote genome for example is very different compared with PGCs, and imprinted regions tend to be protected during this de-methylation process, allowing parent-of-origin-specific gene expression.^97-100^ The level of methylation and the kinetics of de-methylation of the paternal and maternal DNA is very different. The sperm DNA has double the level of methylation (80–90% CpGs) than DNA of the egg (40% CpGs), and is quickly de-methylated soon after zygote formation.^97-99^ On the contrary, the maternal DNA undergoes a delayed, replication-dependent de-methylation.^97-99^ This implies that an efficient active de-methylation mechanism must be in place soon after fertilization and that the maternal DNA must be shadowed to it (Fig. 2). This differential de-methylation required an active TET3 that specifically localizes to the paternal pronucleous inducing a conversion from 5mC to 5hmC as a first step in the de-methylation program.^101^ Since the affinity of DNMT1 for oxidized 5mC (5hmC, 5fC and 5caC see below: A molecular future) is extremely poor, it is also possible that methylation is reduced passively during cell division.^102-104^

The maternal genome in the zygote is protected from the TET3 activity by a protein called STELLA (also PGC7 or Dppa3). STELLA has a strong affinity for the maternally enriched dimethylated histone H3 Lys9-marked chromatin (H3K9me2) and this interaction results in a change of chromatin structure that prevents TET3 binding.^105^ The protection from the rapid de-methylation driven by differential paternal-maternal H3K9me2/STELLA interaction could also be a mechanism to maintain genomic imprinting. In fact, imprinted gene loci undergo a complete loss of methylation if STELLA is not present and some paternally imprinted genes are found to retain H3K9me2-marked chromatin, preserving them from rapid de-methylation post fertilization (Fig. 2).^105^

The maternal DNA sees a passive loss of methylation achieved by nuclear exclusion of DNMT1. The level of methylation of maternally imprinted genes is however preserved and a low level of nuclear DNMT1 is essential to maintain genomic imprinting.^106^ DNMT1 in this context is part of a complex of proteins that has at its center ZFP57 (Krueppel-associated box (KRAB) domain zinc finger protein) and TRIM28 (also KAP1).^107^ Other components of this complex are nucleosome remodelling and histone deacetylation (NuRD), H3K9me3-catalyzing histone methyltransferase (Setdb1), heterochromatin protein 1 (HP1), and DNMT3a/b^108-110^ (Fig. 2). This complex localizes to ICRs, possibly thanks to a recognition site for ZFP57 (TGCCGC).^111^ ZFP57 affinity for this binding site is methylation dependent and increases when the site is methylated.^68,108^ So during early cell divisions the ZFP57/TRIM28 complex, brings DNMT1 to ICRs in a ZFP57 target-dependent manner, enabling the maintenance of DNA methylation during this early stage.^112^ TRIM28 also interacts with HP1 and Setdb1.^113^ Since H3K9me3 associates strongly with DNA methylation, the TRIM28/HP1/Setdb1 complex may participate (via histone modifications) in the preservation of DNA methylation during this early stage of development.

This mechanism may be robust enough to permit even some initial loss of methylation in imprinted regions (as it was observed) as long as it does not affect the ZFP57 target sites.^114,115^ The ZFP57/TRIM28 complex recruit DNMT3a/b that may be able to compensate for the initial loss of methylation.^114,115^ ZFP57 is expressed during embryogenesis, but is restricted to ovaries and testes in the adults^116^ suggesting that ZFP57 is necessary to maintain methylation at imprinted loci during development.

DNA methylation is often associated with histone modifications. We have already encountered 2 of these modifications (H3K9me2–3), but also H3K4me3 and H3K36me3 play fundamental roles in the preservation of DNA methylation in imprinted loci. H3K4me3 for example specifically inhibits DNA methylation and is relegated to the unmethylated allele in imprinted regions.^117^ DNMT3a/b and their co-factor DNMT3L interact to the histone 3 (H3) tail via a ATRX-DNMT3-DNMT3L (ADD) domain. Methylation of H3K4 (H3K4me3) either prevents this interaction (with DNMT3L) or inhibits DNMT3 activity (DNMT3a/b).^118^ The PWWP domain of DNMT3a/b is important for the interaction with H3K36me3, which intriguingly is found to be enriched in gene bodies of active genes.^119-121^ So DNMT3a/b localization to a specific imprinted region depends on the absence of H3K4me3 (H3K4me0) and the presence of H3K9me3 (see above).^122-124^

Therefore, methylation at imprinted loci is maintained thanks to the ZFP57/TRIM28 localization to these regions that results in the preservation of H3K9me3 via Setdb1 and DNMT3a/b recruitment (Fig. 2).

### Behavioral evidence of genomic imprinting in social insects

Differences in worker ovary size and stinging behavior in *A. mellifera* show strong parent-of-origin effects^132,133^ suggesting that an imprinting mechanism may be in place.

It has been extensively demonstrated that honeybee defensive behaviors are heritable in a parent-of-origin dependent manner. Africanised honeybees (*Apis mellifera scutellata*) are known to exhibit a more rapid stinging response to alarm stimuli (visual or pheromonal), that is more sensitive, with a lower threshold before initiating a sting response, compared with the European subspecies.^132^ Through backcrossing and reciprocal cross studies this defensive behavior has been correlated with paternal inheritance. Drones from F1 queens of a cross of Africanised drones and European queens were used for backcrosses in Stort and Goncalves' study which found increased defensive behaviors in backcross colonies.^134^ Guzman-Novoa and Page report workers of a cross with an Africanised paternity have a sting response equal to that of those with full Africanised inheritance.^135^ Backcrossing of F1 gynes twice with European drones resulted in the same worker stinging response as full European control workers. Similarly, DeGrandi-Hoffman and colleagues found that colonies with an Africanised paternity showed a higher level of defensive behaviors regardless of whether the colony was founded by an Africanized or European queen.^136^ Reciprocal crosses by Guzman-Novoa et al. also showed F1 workers with Africanised paternity to have a greater sting response compared with F1 workers with European paternity. F1 colonies with European paternity exhibited a response intermediate to that of control European and Africanised colonies.^137^

If imprinting is present, one would expect these asymmetries in behavioral phenotypes between F1 colonies of a reciprocal cross. These examples of parent-specific behaviors are consistent with the theory of imprinting via increased patrigenic expression or silencing of matrigenic expression. High expression of patrigenic alleles involved in increased aggressive behaviors with conspecifics is likely to promote the lineage of those individuals.^137^ Whereas matrigenic alleles of the same genes are benefited when silenced as it will reduce the costs of increased colony defensive behaviors.

It is also worth noting a non-social, parasitic wasp (*Nasonia vitripennis*) of the same lineage (hymenoptera), also has been reported to show a “grandfather effect” in which F1 females of reciprocal crosses of closely related species show mating behaviors similar to their paternal heritage.^138^

Although the heritability of defensive behaviors has been well recorded, it has been questioned whether there is a sufficient range of empirical studies regarding the effect of imprinting on conflict-resolution behaviors.^139^ Though more recently, other examples of potential behavioral effects of imprinting in social insects have been identified in the reproductive behavior of honey bees^133^ and caste allocation and behavior effects in ants.^140,141^

A reciprocal cross between 2 honey bee subspecies (*Apis mellifera capensis* and *A. m. scutellata*) found F1 workers with an *A. m. capensis* father to produce on average 30% more ovarioles than F1 workers with an *A. m. scutellata* father.^133^ In the wild, *A. m. capensis* workers are unique in the ability to produce reproductive female offspring through thelytokous parthenogenesis.^142^ Hence male *A. m. capensis* individuals may experiencing an increased “motivation” for their worker daughters to produce a greater number of ovarioles and monopolise gyne reproduction. Whereas founding queens are neither benefited or at a disadvantage from worker gyne production.

Parent-of-origin specific effects on caste and behavior have also been reported in ants.^140,141^ Libbrecht et al.^141^ conducted *Linepithema humile* crosses to reveal the proportion of queens and workers produced in a colony as determined by the paternal lineage. The proportions of queens and males, and all females and males were affected by the interaction between parental lineages. *L. humile* ants from the same crosses were also used to demonstrate that the maternal lineage affected the behaviors of efficiency to collect pupae, foraging propensity, and the distance between non-brood-tenders and brood.^140^ Similarly, the paternal lineage influenced 2 other ant behaviors: the efficiency to feed larvae and the distance between brood-tenders and brood. Therefore, differences in parental selection pressures exist, and this observed behavior is highly consistent with the traits of genomic imprinting.

## Parent of origin allele specific expression

### Haig's theory predicts workers reproduction genes should be imprinted

**Figure 3.**
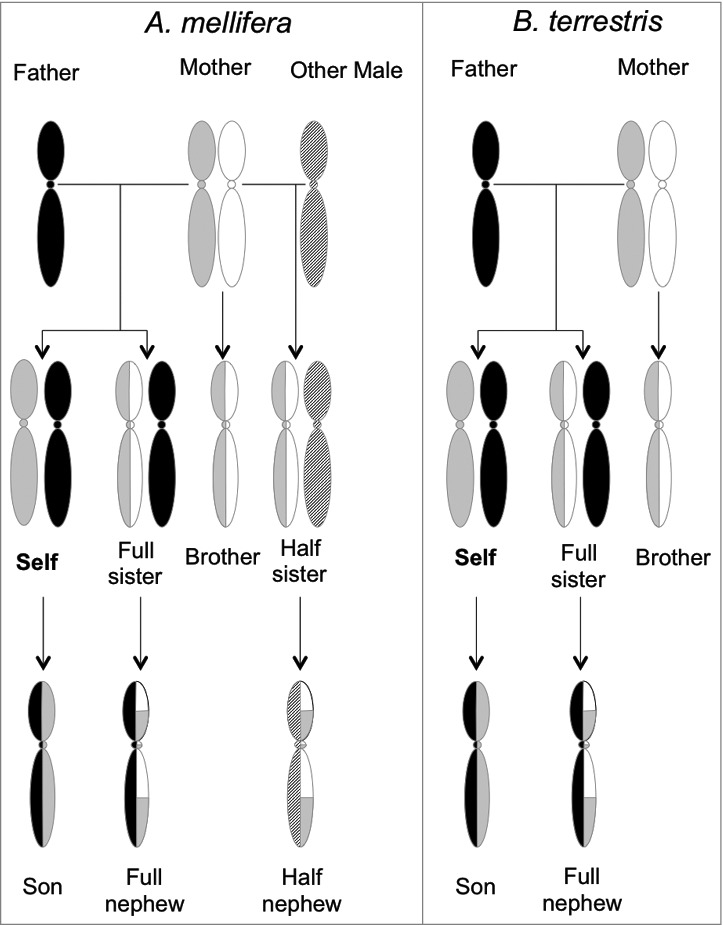
Genetic basis for intragenomic conflict in *A. mellifera* (left) and *B. terrestris* (right). In honey bee colonies a single queen (diploid) mates with multiple haploid males. In bumblebee colonies the queen mates only with a single male. Re-drawn from Queller 2003 and Galbraith et al. 2016.^4,143^

Haig's kinship theory predicts different outcomes in terms of imprinted genes and selfish/selfless behavior depending on the specific social structure considered. Here we outline the theory in relation to 2 important species: *Apis mellifera* (European honeybee), a single diploid queen mated by multiple haploid males and *Bombus terrestris* (Buff-tailed bumblebee), a single diploid queen mated by a single haploid male. We also discuss how the theories predictions change for each species depending on the presence or absence of the queen, referred to as a queenright colony or queenless colony respectively (Fig. 3).

Why should a worker (self; Fig. 3) take care of her sisters instead of reproducing? In haplodiploid species females hatch from fertilized (diploid) eggs, while males hatch from unfertilized (haploid) eggs, so the degree of relatedness between offspring is different for matrigenes and patrigenes. In the case of bumblebees, the coefficient of relatedness between self (Fig. 3 right) and other sisters is r:3/4 (they share 3/4 of their genome on average), which is higher than their relatedness with their own offspring (r:1/2), so this may explain their selfless behavior.

These coefficients of relatedness between bumblebee sisters or toward offspring change when calculated independently from the perspective of a patrigene and a matrigene. For a patrigene r:1 since all sisters have the same father, while r:1/2 for a matrigene. In addition, for patrigenes in self, r:0 with any males produced by the queen. So in a queenright bumblebee colony, the only chance for the self patrigene to be passed to the next generation is to favor self reproduction or reproduction by her sisters (either workers or new queens) at the cost of rearing fewer brothers. Matrigenes however should be selected to regulate self reproduction when it is associated with high costs to brothers as these genes are less likely to occur in nephews than they are in brothers.

However, should the singly mated bumblebee queen die and the self begin to reproduce, a patrigene would have the same probability to be passed to the next generation either by self or self's sisters (r:1/2). Patrigenes should now not experience selective pressure for reproductive behavior, meaning sterility and nursing behavior are just as beneficial as reproductive behavior. For a matrigene the situation is different since this probability is reduced (r:1/4) in the self's sisters. So under queenless conditions with self reproducing, matrigenes may be imprinted to promote reproduction and avoid sterility.

In honeybee colonies queens mate with multiple males, so a worker (self; Fig. 3 left) has both full sisters and half sisters. The coefficient of relatedness between self and sisters varies between r:3/4 for full sisters and r:1/4 for half sisters. From a patrigene perspective r:1 with full sisters but r:0 with half sisters because the latter have a different father. To a matrigene half sisters and full sisters looks the same having both r:1/2. The matrigene in a worker has an equal (50%) chance of being in a brother as well as in self's offspring. Therefore, the matrigene tries to ensure an equal distribution of her resources among both her sons and daughters. In a queenright situation once again patrigene may promote self reproduction because this is the only way to be passed to the next generation. In a queenless situation self should be selected to begin to reproduce, since the patrigene (with no relatedness to half sisters and their progeny) will only increase its fitness if self, or full sisters, reproduce. On the contrary, matrigenes having the same degree of relatedness with both full and half nephews (r:1/4) should be selected to moderate reproduction if this comes at a high cost to half sisters. So in the case of honey bees when a queen dies and self begin to reproduce, patrigenes may been imprinted to promote reproduction and avoid sterility.

Haig's kinship theory provides the theoretical basis to predict genomic imprinting in hymenopteran eusocial insects (e.g., *A. mellifera, B. terrestris, C. floridanus*). These insects have full epigenetic toolkits including DNMTs and TETs, they display complex social structures with caste specific behaviors, and differences in matrigene-patrigene relatedness due to the haplo-diploid sex determination, which are all elements that reinforce the expectation for genomic imprinting. So, is there any evidence for genomic imprinting in hymenoptera?

### Evidence for parent of origin allele specific expression

Testing candidate genes using allele-specific (parent-specific) amplifications, Amarasinghe and colleagues^145^ identified 2 genes (*ecdysone 20-monooxygenase-like* and *IMP-L2-like*) with parent-of-origin specific expression in *B. terrestris*. The pattern of expression was consistent with the prediction of Haig's kinship theory that genes associated with initiation of worker reproduction should be paternally expressed in queenright *B. terrestris* workers, and genes that inhibit reproduction should be maternally expressed. Indeed, *Ecdysone 20-monooxygenase-like*, a gene involved in ovary activation,^146^ was paternally expressed and *IMP-L2-like*, that was implicated in inhibition of worker reproduction,^147^ was maternally expressed. This expression profile was evocative of a possible genomic imprinting mechanism at work.

**Figure 4.**
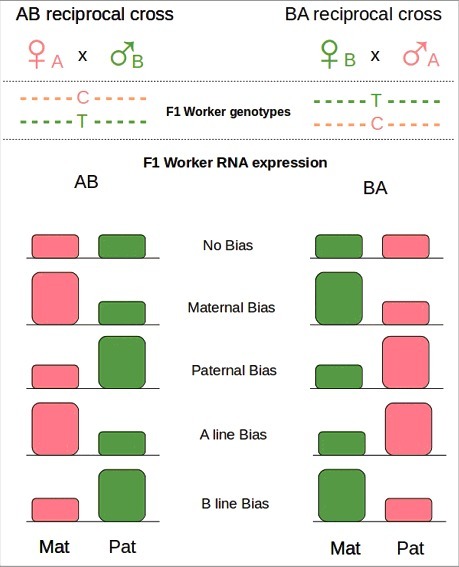
Reciprocal crosses. Females from different lineages (A and B) are crossed reciprocally with single drones of the opposite lineages (B and A). Because of SNPs between the lineages maternal and paternal alleles are recognizable in F1. The global level of expression of maternal (Mat) and paternal (Pat) alleles in a tissue can be tested by RNA-seq. Parental Bias results in an overexpression of the Parental allele (Mat or Pat) in both crosses. Lineage Bias results in an overexpression of the lineage specific allele (A or B). Redraw from Kocher et al 2015.^144^

Recently a few studies have looked for parent-specific gene expression (PSGE), possibly due to genomic imprinting, with interesting results in the honeybee *A. mellifera*.^143,144^ These studies took advantage of reciprocal crosses to investigate the prediction that PGSE should affect reproductive and cooperative behaviors of non- reproductive workers (Fig. 4). This approach uncouples parent-of-origin effects from lineage-specific effects, generating single drone insemination between 2 different lineages. In Kocher et al. 2015 the authors used 2 different lineages from Europe (*Apis mellifera carnica*) and Africa (*A. mellifera scutellata*) while in Galbraith et al. 2016 they used *A. mellifera ligustica* from both continents. Crucially for the identification of any PSGE, the lines used showed behavioral differences and most importantly they had many single nucleotide polymorphisms (SNPs) that allowed identification of maternal and paternal alleles.^148^ In Kocher et al. 2015, the authors identified only a small group of significant PSGE, mostly maternally biased, while in Galbraith et al. 2016, the authors reported an association between patrigene expression and worker reproduction (Table 1). In Kocher et al. 2015 the parental allele expression was compared in queen right guard worker bees (full body), larvae (full body), and individual forager brains. They found 12 (larvae), 17 (guards), and 23 (brains) maternally biased genes and 3–4 (guard brains) paternally biased genes. In this environment (queenright) one may expect to find imprinted genes that repress worker reproduction and mediate social behavior (perhaps reducing aggression). The adult tissues where it is more likely that genomic imprinting would result in PGSE could reasonably be the ovary (being directly affected by changes in reproduction) and the brain (mediating reproduction related behavior). Indeed one of the genes in the brain with PGSE was *huntingtin* that has been associated with stinging behavior,^137^ and a maternally expressed gene in larvae (*Neural Lazarillo*) that downregulates insulin signaling, a pathway important for queen-worker differentiation.^149^

**Table 1.** Comparison of PSGE in 2 *Apis mellifera* studies, Kocher et al. 2015 and Galbraith et al. 2016.

	Kocher et al. 2015	Galbraith et al. 2016
Same subspecies	No (*A. mellifera carica* and *A. mellifera scutellata*)	Yes (*A. mellifera ligustica*)
Tissue	Brain/full body	Fat body and ovaries
PSGE	Mostly maternally biased expression	Mostly paternally biased expression
Association between PSGE and methylation	No	No

**Table 2.** Useful Technologies for addressing questions of mechanism of GI.

Name	Target /Use	Characteristic	References
RNA-Seq	RNA sequence small RNA sequence	Returned expression levels and sequence of RNA	^198^
			^199^
	Difference in expression	Accurate quantification	
	Alternative splicing		
BS-Seq	Identify 5hmC	Single base resolution	^200^
		Accurate quantification	^201^
		Do not discriminate between 5mC and 5hmC	
TAB-Seq oxBS-Seq	Identify 5hmC	Single base resolution	^162^
		Discriminate between between 5mC and 5hmC when paired with BS-Seq	^163^
redBS-Seq	Identify 5hmC	Single base resolution	^164^
		Discriminate between between 5mC and 5hmC when paired with BS-Seq	
ChIP-Seq	Identify DNA-protein interactions	Returns maps of DNA binding for proteins of interest	^202^
		Requires good quality antibodies	^203^
iChIP	Identify DNA-protein interactions	Returns maps of DNA binding for proteins of interest	^183^
		Requires good quality antibodies	
HT-ChIP	Identify DNA-protein interactions	Returns maps of DNA binding for proteins of interest	^184^
		Requires good quality antibodies	

For a recent review on other omic technologies see^204^

Most likely a combination of different reasons explained the small number of maternally biased genes identified in Kocher et al. 2015. For instance no particular phenotypic or behavioral difference was imposed on the bees tested. Moreover, full bodies were used for larvae and guards, masking any possible tissue specific effects (even though they used forager brains with similar results)(Table 1). But most importantly they suffer from a strong lineage-of-origin bias that would cloud any other allele-specific bias. In fact, they suggest that mito-nuclear incompatibilities in the hybrids were affecting the results, and more recently they expand on that observation confirming it.^150^

In the paper by Galbraith and colleagues, the authors took advantage of a clear phenotypic and behavioral difference between African and European bees where African workers tend to have larger ovaries and become more readily reproductively active^151^ (Table 1). These differences allow the kinship theory prediction that patrigenes should promote reproduction to be tested. In the reciprocal crosses a strong patrigenic effect was evident at the phenotypic level. In fact workers from the reciprocal crosses with African fathers showed an increased number of ovarioles and were more likely to become reproductive compared with workers with European fathers. To test for PSGE, workers from the reciprocal crosses were maintained in an environment (queenless and broodless) that was inducive to reproduction, and difference in expression was measured in ovaries and fat bodies of reproductive and non-reproductive individuals. In this condition one would expect that regardless of the lineage-of-origin of the father, patrigenes should be upregulated in both non-reproductive and reproductive workers but even more so in the latter. Of the 303 identified transcripts with an allele bias, the number of patrigenes over represented in sterile and reproductive workers was 143 and 181 respectively with an additional 12 and 13 matrigenes. These results broadly confirmed the initial predictions. Genes of the ecdysone-vitellogenin pathway, controlling egg production, including *vitellogenin* (that encode for a yolk precursor protein), showed paternal expression. Some genes that were paternally biased only in reproductive workers included *yolkless, ecdysone receptor* (*ECR*) and *ecdysone-induced protein 75* (*E75*).

Comparing transcription of ovaries between reproductively active and inactive individuals could include an intrinsic source of noise. In fact, although oocytes are not transcriptionally active and the on-set of zygotic transcription is usually placed after egg deposition (5 h after egg laying in the wasp *N. vitripennis*^152^), the oocytes include several maternally transferred mRNA (for a review see^153^) that may not be represented in tissue from non reproductive individuals.

Both papers provide evidence for parent-of-origin effects in the progeny, but they offer very different results. This may suggest that PSGE is conditional to the specific context in which the difference in expression is tested, implying that different genes may be parentally imprinted in different cell lineages. PSGE may therefore be revealed only in specific environmental contexts. For example, an imprinted gene promoting ovary development, may result in a difference in expression solely when a worker becomes reproductive in a queenless, broodless situation and in a specific tissue (e.g., ovary). In the same animal a gene may be imprinted in one tissue and not in another, and a difference in expression becomes visible only in a tissue-specific, condition-specific manner.

How parental allele bias is achieved is still unknown but some interesting observations can be drawn from these papers. In mammals, genomic imprinting is often associated with DNA methylation but Kocher et al. 2015 did not find any association between their parentally biased genes and known methylated genes (Table 1). We compared Galbraith et al. 2016 list of PSGE with known methylated genes (taken from^67,34,31^ and^49^), not only did we not find a correlation between the 2, in fact there were zero genes in common between the 2 lists. These findings have also been mirrored in,^154^ where only a small list of genes were found to overlap between Galbraith et al. 2016 PSGE list and gamete/embryo methylated genes from.^155^ It is entirely possible little overlap is seen because the samples between these studies are too varied, PSGE and methylation may differ depending on tissue and developmental stage. When investigating the role of methylation in PSGE it is worth noting epialleles, where the sequence itself determines methylation status, can potentially be confused for parent-specific methylation.^156^ It is vital that future studies, exploring methylation as a regulatory mechanisms for genomic imprinting in hymenoptera, take this potential confusion into consideration. However, in hymenoptera, methylation is often found in gene bodies marking intron-exon junctions and is associated with splicing more than gene silencing. Chromatin remodelling in the fruit fly *Drosophila melanogaster* was associated with parent-of-origin expression^11^ and both Kocher et al. 2015 and Galbraith et al. 2016 identified genes involved in this process. So it is quite possible that other epigenetic mechanisms (e.g., histone modifications) are generating the genomic imprinting that provoke the PSGE observed in these papers.

## A molecular future: Prospects

Next generation sequencing technologies have permitted an extraordinary advance in our understanding of the genetics of many hymenoptera species in a relatively short time. RNA-seq and sequencing of bisulfite converted DNA (BS-seq) are powerful tools enabling researchers to ask and answer new and important questions about the distribution and functions of DNA methylation in hymenoptera as well as to test the prediction of Haig's kinship theory. As noted above careful analyses of these data are required, epialleles^156^ and poor statistical analysis^64^ could lead to genomic imprinting being incorrectly labeled. In fact Wedd et al. 2016 describe a differentially methylated epiallele (of the gene *AmLAM* ) in the honeybee, where an increase in methylation correlated with an increase in gene expression, but only in certain developmental stages and tissue types.^157^ This shows how important it is to take into account epialleles, splice forms, tissue type and developmental stage when exploring methylation as a mechanism for genomic imprinting in insects.

Recently other modifications on cytosines have been described in many tissues in mammals including 5-hydroxymethylationcytosine (5hmC), 5-formylcytosine (5fC) and 5-carboxylcytosine (5caC). TET enzymes can convert 5mC to 5hmC and further oxidise it to 5fC and 5caC in a possible demethylation pathway back to unmodified C.^38,158,159^ 5hmC in addition may be an epigenetic mark on its own^160^ for example changing the DNA interaction dynamics of 5mC binding proteins and functioning as an off-switch.^161^ The identification of intermediate C modifications may therefore be important in the context of rapid re-programming associated with response to environmental changes, tissue specific epigenomes, caste specification, and genomic imprinting. BS-sequencing cannot discriminate between 5mC and 5hmC because both are resistant to deamination by sodium bisulfite treatments so until recently whole genome quantification of 5hmC has been very difficult. New technologies however are now available to map both 5hmC and 5fC at a genome level with single base resolution.^162-164^ Tet- assisted bisulfite sequencing (TAB-Seq) and oxidative bisulfite sequencing (oxBS-Seq) combined with BS-seq has allowed mapping of full genome 5hmC at single-nucleotide resolution,^162,163^ while reduced bisulfite sequencing (redBS-Seq) permits identification of 5fC with the same resolution.^164^ To increase their statistical and biologic power, future experimental design should consider carefully both number of independent biologic replicates, tissue specificity as well as developmental and social context.

Although DNA methylation seems to have some conserved functions between mammals and hymenoptera (e.g., control of splicing) it is not clear whether its role in genomic imprinting is shared by the 2 groups. Genomic imprinting is widely found in mammals^165^ and plants,^166^ and imprinted chromosomal regions have also been identified in insects,^167^ fish^168^ and nematodes.^169^ The epigenetic markers that are most frequently associated with genomic imprinting are histone modifications. Methylation of some of histone 3 (H3K9, H3K27, H3K4) and 4 (H4K20) lysines are among the most common modifications associated with genomic imprinting.^170^ In particular H3K9me is a shared feature of imprinting in mammals (see Box 1, Genomic Imprinting: the mammalian way), plants (*A. thaliana*)^171^ and insects (*Planococcus citri* ).^172^ Even in *Drosophila*, where only a small fraction of the genome shows any methylation at all (0.5% in early embryo^173^), both H3K9 and H3K4 methylation are associated with genomic imprinting^11,174^ suggesting that these modifications may be part of an ancestral common mechanism. Chromatin immunoprecipitation sequencing (ChIP-seq) is a versatile technology that identifies genome wide DNA-protein interactions^175^ and has been successfully used to investigate histone modifications^176^ and chromatin remodelling complexes^177^ as well as RNA polymerase and transcription factor binding regions.^178,179^ This approach used in the context of hymenoptera species could provide key answers regarding changes in chromatin structure and histone modifications related to different development stages, caste definition, and possible mechanisms of genomic imprinting. A limitation of this approach is that it requires a large sample size (1–10 million cells) that could be problematic to achieve especially in the context of environment-specific/tissue-specific epigenetic re-programming.^175,180^ Nevertheless, both ChIP and ChIP-seq have been successfully used in *D. melanogaster*,^181182^ and recent advances in this technologies such as indexing-first ChIP (iChIP)^183^ and high- throughput ChIP (HT-ChIP)^184^ dramatically reduce the amount of cells required and could be adapted to work in insects.

CRISPR gene editing is one of the most exciting and far reaching tools available today to the molecular biologist for manipulation of target genes. CRISPR has been successfully used in a variety of insect species including Diptera (*Drosophila melanogaster, Anopheles gambiae, Aedes aegypti*),^185-187^ Coleoptera (*Tribolium castaneum*),^188^ Lepidoptera (*Bombyx mori* ),^189^ and lately hymenoptera (*Nasonia vitripennis, Apis mellifera, Ooceraea biroi* ).^190-192^ Due to differences in life cycle, social structure, and ease of laboratory manipulation, some hymenoptera species may be more suitable than others to support CRISPR approaches. At the very least this technology could be used to induce knockout or mutations of genes suspected to be involved in genomic imprinting (e.g., DNMTs, TETs, Histone modification proteins), but it has also the potential to facilitate the functional investigation of protein domains and control target expression. Mimicking an approach that has been already tested for mammalian DNMTs,^193^ CRISPR could facilitate the generation of transgenic flies (*D. melanogaster*) expressing hymenoptera genes (e.g., DNMTs, TETs, Histone modification proteins) in null backgrounds, therefore enabling their functional characterization, contributing greatly to our understanding of the mechanism of genomic imprinting. The functional characterization of DNMTs could also help to understand why hymenoptera and mammals differ so dramatically in their level of CpG methylation. A possibility is that the “common” status of hymenoptera DNMTs is to be functionally/molecularly inactive or in a “closed” conformation and require a cofactor(s) to be “open” and/or active. Alternatively, an unknown cofactor(s) may strongly repress DNMTs limiting their activity to specific sites. In addition, other epigenetic mechanisms (e.g., histone modifications) may contribute to the observed CpG methylation difference between mammals and hymenoptera, making DNA more or less accessible to DNMTs, implying in fact that the majority of hymenoptera DNA is unavailable at any given time/tissue. CRISPR may also permit the implementation of DNA adenine methyltransferase (Dam) identification (DamID)^194^ a powerful technology that has been used for the identification of chromatin interacting proteins and other DNA-protein interactions (see Pindyurin et al., 2016^195^ for a recent DamID application in *D. melanogaster*). Recently a very exciting application of CRISPR-Cas9 technology has been used to manipulate the level of methylation of specific cytosines in a target specific manner. Mammalian cells were transformed with a catalytic inactive form of Cas9 fused with either TET1 or DNMT3a along with gRNAs that direct enzymatic activity in a sequence specific manner.^196,197^ This technology, adapted to be used *in vivo* in hymenoptera, will be a powerful tool to study the function of specific CpG methylation and the contribution of DNA methylation to genomic imprinting, splicing, caste definition, tissue specificity, and much more.

Using a combination of these technologies in a hypothesis driven context (e.g., Haig's kinship theory predictions) will allow us to explore the mechanism of genomic imprinting in hymenoptera. Moreover, it will be possible to explore commonalities as well as differences between hymenoptera and other insects, mammals, and plants in the mechanism of genomic imprinting.

## Closing remarks

The quest for understanding genomic imprinting and the mechanisms that generate and maintain it in hymenoptera is only beginning. Although many questions are still unanswered, some relevant observations are already possible. Genomic imprinting may be not a common characteristic to all hymenoptera since evidence are lacking for some species (e.g *N. vitripennis*) and may be more common in eusocial insects as initial investigation in *A. mellifera* suggests. Genomic imprinting may not have a binary status but be very much a variable of time (developmental time), social context (e.g., caste definition, queenright or queenless), tissue, and physiologic status. By its very nature, the epigenetic code (including DNA methylation, C intermediate status, histone modifications, and more) is plastic and responsive to environmental changes, tissue specific, and probably variable during development. So it is not far fetched to suppose that also genomic imprinting, relying on epigenetic modification for its generation and maintenance, could be as plastic. The definition of a specific context (e.g., tissue specificity, social context, age) is particularly important for studies that apply an expression only approach. A poor choice of tissue and or social context may produce results of difficult interpretation. Future work therefore will be facing the challenge of defying with particular care the context of genomic imprinting investigation and should combine multiple technologies to investigate differences in expression as well as changes in epigenetic markers.

Whether DNA methylation is associated with genomic imprinting in hymenoptera is still an open question; however, its role in controlling splicing has been supported in different species in many studies. Alternative splicing could be a possible target of genomic imprinting where the expression of a splicing variant is parent-of-origin specific. Examples of sex specific alternative splicing are found both in the fruit fly *D. melanogaster*^205^ and mammals^206^ and DNMT1 alternative exon splicing results in maternal/paternal differential expression in mammalian germ cells.^207^ In hymenoptera, DNA methylation could play a crucial role mediating the inclusion/exclusion of introns/exons in a parent-of-origin specific manner determining the parent-of-origin variant expression. The investigation of other epigenetic markers including intermediate C modifications and euchromatin signals (e.g., histone modifications) is becoming crucial for the understanding of the mechanism of genomic imprinting. It is now fundamental to identify the proteins mediating genomic imprinting as well as defining their molecular functions. This task as challenging as it is, may be facilitated by the availability of technologies such as CRISPR and Chomatin IP that allow us to explore the role of target genes as well as whole genomes. The vast amount of knowledge from mammals, plants, and *Drosophila* regarding the genes and histone modifications involved in genomic imprinting as well as the resources already available for these species, could serve as a vantage starting point for the challenges ahead.

Is it possible to generate and maintain gene imprinting without DNA methylation in hymenoptera, and what is the role of H3K9 and H3K4 methylation and other histone modifications? What is the relation if any between 5mC, 5hmC and 5fC and gene imprinting/alternative splicing? Is alternative splicing a target of genomic imprinting? What are the proteins mediating genomic imprinting? These are but few of the questions that need to be answered to understand the mechanism of genomic imprinting in hymenoptera.

The studies today available^143,144^ suggest that genomic imprinting is present in the honeybee *A. mellifera* but in a very complex interaction between several variables. Questioning the prediction of Haig's kin theory in other species has become now essential to reveal whether it is a common strategy in eusocial insects. Will genomic imprinting be pervasive in other eusocial insects (e.g., *B. terrestris, C. floridanus*)?

We think that we are now at the dawn of a very exciting time having the extraordinary opportunity to dissect the epigenetic code and genomic imprinting with unprecedented detail and from both mechanistic and evolutionary points of view using population genetics and molecular biology approaches. This will provide answers to many of today's questions and hopefully allow us to ask many more.
